# Enhancing Cognitive Functions in Older Adults With Mild Cognitive Impairment via Virtual Sail 3D: Protocol for a Feasibility Randomized Controlled Trial

**DOI:** 10.2196/85089

**Published:** 2026-01-15

**Authors:** Federica Sancassiani, Alessandra Perra, Veronica Vacca, Roberta Montisci, Mariarita Monni, Stefano Lorrai, Giulia Cossu, Donatella Rita Petretto, Lorenzo di Natale, Diego Primavera, Stefania Redolfi, Angelo Scuteri, Antonio Egidio Nardi, Goce Kalcev, Massimiliano Pau, Mauro Giovanni Carta

**Affiliations:** 1 Department of Medical Sciences and Public Health University of Cagliari Cagliari Italy; 2 University Hospital of Cagliari (AOU Cagliari) Cagliari Italy; 3 Department of Pedagogy, Psychology and Philosophy University of Cagliari Cagliari Italy; 4 Cerebrum VR Society Rome Italy; 5 IDEGO Digital Psychology Society Rome Italy; 6 Laboratory of Panic and Respiration-IPUB Institute of Psychiatry of the Federal University of Rio de Janeiro Rio de Janeiro Brazil; 7 Department of Mechanical, Chemical and Materials Engineering University of Cagliari Cagliari Italy

**Keywords:** aging, cognitive functions, emerging technologies, feasibility, mild cognitive impairment, Sail, virtual reality

## Abstract

**Background:**

Innovative approaches, such as virtual reality (VR)–based cognitive rehabilitation, are becoming essential to healthy aging.

**Objective:**

This study aims to evaluate the feasibility and generate preliminary evidence regarding the effectiveness of an immersive VR-based cognitive remediation intervention for older individuals with mild cognitive impairment (MCI).

**Methods:**

A total of 40 individuals aged 65 years and older, of both genders with MCI, will be recruited and randomly assigned to the experimental or control group. The experimental group will participate in a 6-week VR-based cognitive remediation program, while the control group will receive healthy lifestyle education. Feasibility will be assessed through measures, including dropout rates, side effects, and satisfaction with the program. Preliminary effectiveness will be evaluated using assessments of cognitive functions, quality of life, functional mobility, depression and anxiety symptoms, regulation of social and biological rhythms, body awareness, and physical activity levels.

**Results:**

Recruitment began in May 2024 and is expected to conclude by November 2025; to date, 153 participants have been recruited and screened, with 39 enrolled. Preliminary analyses are planned for January 2026 and follow-up analyses for January 2027.

**Conclusions:**

This trial will provide insights into the feasibility and preliminary effectiveness of a VR-based intervention for older adults with MCI, with implications for its integration into preventive and rehabilitative care.

**Trial Registration:**

ClinicalTrial.gov NCT06579378; https://clinicaltrials.gov/search?term=NCT06579378

**International Registered Report Identifier (IRRID):**

DERR1-10.2196/85089

## Introduction

With the ongoing global increase in life expectancy, mild cognitive impairment (MCI) and dementia have emerged as leading causes of disability, especially among older adults [1]. Italy has the highest proportion of older adults in Europe, with 23% of its population aged 65 years and older [2]. Projections suggest that by 2050, 1 in 5 Italians will be in advanced age [2]. This demographic shift is expected to result in a shrinking working-age population, accompanied by a significant increase in both direct and indirect health care and social costs associated with age-related health issues [3]. MCI is characterized as a transitional phase bridging the gap between typical cognitive decline and the early stages of dementia [4]. Cognitive decline is frequently associated with symptoms of depression and anxiety, both of which contribute to a progressive deterioration in functional capacity during daily activities [5]. These conditions negatively impact quality of life (QoL) and disrupt the regulation of biological and social rhythms [6]. Given these considerations, both researchers and the European Union have increasingly focused attention on the critical objective of advancing research in the field of healthy aging, particularly in the development and implementation of nonpharmacological interventions aimed at preventing or delaying cognitive decline [7,8].

Emerging evidence from recent literature reviews highlights the beneficial impact of cognitive training, cognitive remediation interventions, and physical activity on cognitive performance in older adults, especially when these approaches are embedded within a comprehensive public health framework [9-11]. Research on active aging has been identified as a priority, particularly concerning the feasibility and effectiveness of nonpharmacological interventions aimed at preventing and/or delaying cognitive decline [11-13]. Risk factors such as physical, cognitive, and social inactivity are estimated to account for about 40% of the risk of developing dementia in a lifetime [14]. Contemporary reviews have shown that cognitive remediation interventions are effective in improving cognitive performance in older adults with and without cognitive decline [15,16]. Furthermore, cognitive remediation is a behavior-based training intervention aimed at improving cognitive functions (memory, attention, executive functions, social cognition, and metacognition) to achieve lasting results and their generalization [17]. There is substantial evidence supporting the effectiveness of such interventions in neuropsychological disorders, such as dementia, MCI, and behavioral disorders [18].

In recent years, there has been an increase in the use of immersive virtual reality (VR) software in clinical and rehabilitative settings as tools for rehabilitation and the improvement of various skills [18,19]. To date, there are few studies where immersive VR is used to enhance cognitive functions related to personal and social functioning, especially to prevent cognitive decline in older adults (aged 65 years and older), and the quality of evidence is rated as moderate to low [20,21]. VR serves as a core tool in facilitating cognitive and functional skill acquisition or enhancement, owing to its capacity to deliver immersive, ecologically valid, and highly realistic experiential learning environments [22]. These findings highlight not only improvements in specific cognitive domains, such as memory, language, and executive functioning, but also improvements in overall clinical, personal, and social functioning [20,21,23]. Despite the encouraging evidence, the methodological rigor of existing studies remains limited. Many studies lack comprehensive descriptions of the VR interventions and the specific methods used during the sessions. Additionally, most studies do not clarify the framework used to develop the interventions, which is essential for understanding the alignment between the hypotheses, outcomes, methods, and the VR programs designed to target specific cognitive domains. This lack of detailed information prevents the establishment of a standard operating procedure, which is crucial for ensuring the reproducibility of the intervention and for guiding the development of a future gold standard in the field [20,21,23-27]. Moreover, an integrative, multidisciplinary approach, grounded in individualized recovery-oriented objectives, is imperative for addressing the multifaceted and evolving needs of people with MCI.

The trial aims to assess the feasibility (primary outcome) of a 6-week intervention to improve cognitive functions using the immersive VR software “CEREBRUM” (Cerebrum VR Society) with sailing scenarios among the older adults with MCI. It also aims to evaluate, through a randomized controlled trial (RCT) design, the effectiveness of the experimental intervention in enhancing cognitive functions and/or mitigating further cognitive decline and deterioration (secondary outcome). Finally, it will verify whether the experimental intervention has a positive impact on (1) QoL, (2) functional mobility, (3) depressive and anxiety symptoms, (4) social and biological rhythms, (5) body awareness, and (6) physical activity levels (tertiary outcomes).

## Methods

### Study Design

**Figure 1:**
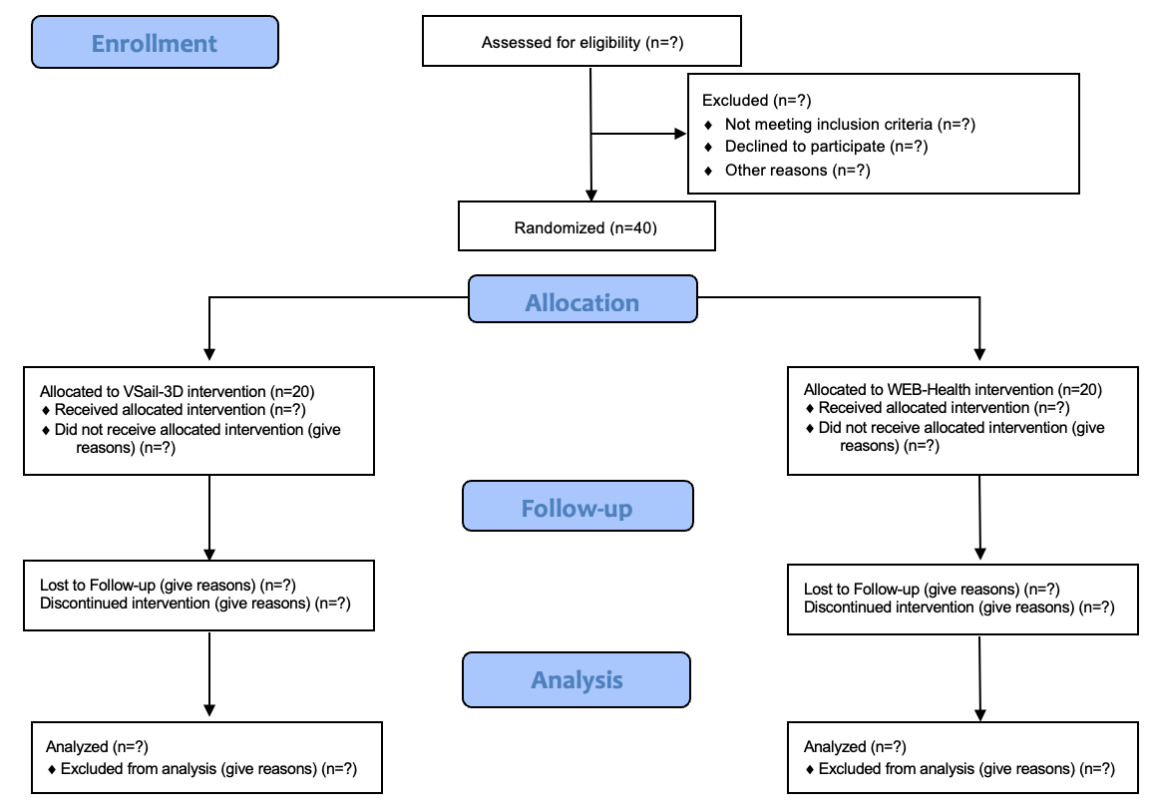
CONSORT flow diagram.

This study is designed as a feasibility RCT. Assessments will be conducted at the following time points: T0 (0 weeks - baseline), T1 (6 weeks), T2 (12 weeks from T1), T3 (24 weeks from T1), T4 (36 weeks from T1), T5 (48 weeks from T1). The study adopted a longitudinal, 2-arm design, with participants randomized to either an active interventional protocol based on the Virtual Sail 3D (VSail) experimental protocol or to a control setting, “WEB-Health” (education on healthy lifestyles to promote active aging). Participants will be independently randomized in a 1:1 ratio to one of the two parallel assignment groups: (1) VSail or (2) WEB-Health. The study protocol is written according to the CONSORT (Consolidated Standards of Reporting Trials) 2010 statement for randomized pilot and feasibility studies [28] (Figure 1).

### Participants, Recruitment, and Setting

The target population includes older participants with MCI conditions.

Inclusion criteria are being 65 years and older of both genders, without conditions that would prevent them from participating in the activities of the experimental intervention “VSail,” without severe issues with autonomous mobility, with MCI according to Addenbrooke’s Cognitive Examination-Revised (ACE-R) for cognitive functions, which also includes the Mini-Mental State Examination (MMSE) [29], with the following ranges: ACE=66.93-84.93 and/or MMSE=27.02-27.11, and signing the informed consent.

Exclusion criteria are severe cardiovascular conditions, severe issues with autonomous mobility, severe metabolic disorders not pharmacologically compensated, severe neurological conditions that prevent from participating in the experimental protocol, such as a stroke within the past 2 years, Parkinson disease, epilepsy, or dementia (Alzheimer, vascular, etc), severe ongoing bronchopulmonary disorders, severe ongoing renal disorders, glaucoma, retinal detachment, or other serious vision conditions that do not allow the safe use of VR technology, active malignant neoplasm that do not allow the hinders participation in the intervention.

Recruitment activities will be conducted at the sites of the project’s partner units, namely, the Consultation-Liaison Psychiatry and Psychosomatics Center, the Cardiology–CCU–Hemodynamics Unit, and the Multidisciplinary Sleep Medicine Center of the University Hospital of Cagliari (AOU Cagliari), Italy. At each site, infographics will be displayed and informational brochures about the project will be made available so that outpatients attending the relevant clinics can express their interest in participating. In addition, a health care professional will contact by telephone all patients who, based on the study’s inclusion and exclusion criteria, will be identified by clinicians of the partner units as potentially eligible. All patients who express interest and meet the eligibility criteria will then be invited to provide written informed consent and to undergo screening with the ACE-R [29].

Participants who screen positive for MCI will be enrolled and subsequently randomized to the experimental or control arm. Those who screen negative will be excluded from study participation.

According to the inclusion and exclusion criteria, recruitment will continue until the target sample size is reached, allowing for randomization and the conduct of interventions.

All assessment activities using the instruments specified in the protocol, as well as the implementation of both the control and experimental interventions, will take place at the Consultation-Liaison Psychiatry and Psychosomatics Center. These activities will be conducted by mental health professionals who will be duly trained in the use of the assessment tools and in the delivery of the VR-based cognitive remediation protocol (experimental intervention) and the web-based healthy lifestyle seminars (control intervention). Motor performance assessment (balance, gait, and functional mobility) will be carried out with the support of biomechanical engineers who will receive appropriate training for this purpose.

### Randomization and Allocation Concealment

Participants who meet the eligibility criteria will be randomly assigned to one of the two groups: an intervention group and a control group. Randomization will be conducted after baseline assessment. Randomization will be computer-based and concealed. The individuals conducting the randomization procedure will be blinded to participants’ identities and statuses and will not receive any information about them. Participants will be randomly assigned to experimental or control groups in a 1:1 ratio using block randomization. Allocation codes were concealed.

### Blinding

Due to the intervention method, it may not be feasible to effectively blind both the participants and professionals engaged in the study.

### Interventions

The control intervention encompasses web-based activities aimed at promoting healthy lifestyles for active aging (WEB-Health). This intervention includes education on healthy lifestyles to promote active aging (topics: physical activity in old age, effective communication, stress management, technologies, and health). It consists of 3 seminars, delivered in the form of webinars, on a biweekly basis over 6 consecutive weeks, lasting 50-60 minutes each. Each webinar will be led by a qualified mental health professional and delivered live in plenary session through the Zoom platform (Zoom Video Communications, Inc).

The experimental intervention (VSail) includes 12 sessions (2 times a week, for 6 consecutive weeks) with scenarios (virtual environments) dedicated to the sport of sailing. The duration of each session is 50-60 minutes. It is based on the immersive VR software “CEREBRUM” developed by the Cerebrum VR Society. The software represents one of the most recent tools in psychiatric rehabilitation, specifically designed for cognitive remediation. Developed by mental health professionals, “CEREBRUM” is compatible with the “Oculus Quest” VR headset, which carries the CE marking, ensuring adherence to European safety, health, and environmental protection standards. Within the immersive virtual environment, participants interacted with scenarios replicating daily activities in both home and urban settings.

For the present project, the “CEREBRUM” software was further developed through the addition of 4 virtual scenarios specifically designed around the sport of sailing. The decision to add these scenarios was driven by the aim of making the virtual experience during cognitive training more interactive and of simultaneously engaging motor functions. The “CEREBRUM” sailing-based scenarios enable users to move within the virtual space to grasp and manipulate objects (eg, the helm) and to perform specific movements required for executing sailing manoeuvres (eg, approaching the sheet, bending down to reach it, and alternating rapid arm movements to haul the line and adjust the sail). The “CEREBRUM” software comprises exercises of varying difficulty designed to train various cognitive functions, including attention, memory, learning, cognitive estimation, working memory, executive functions, motor abilities, and language. The different degrees of difficulty are designed to adapt to the user’s functional diagnosis. The operator who conducts the intervention must adapt the difficulty level to the user’s residual abilities so that the exercises are neither too easy nor too complex. Each session, after an initial part of welcome, psychoeducation and orientation to the instrument, involves alternating VR exercises for a maximum of 15-20 minutes, positive and corrective feedback, and suggestions of practical homework related to the cognitive function trained during the session that the individual should try during daily life (generalization phase).

### Outcomes

The primary outcomes regard feasibility and include (1) dropout rates, (2) the proportion of recruited participants among those considered eligible, (3) the incidence of potential adverse effects associated with the use of immersive VR technology (eg, nausea, dizziness, visual fatigue, and headache), and (4) the level of perceived satisfaction reported by participants following the experimental intervention.

The secondary outcomes regard cognitive functioning and include (1) executive functions, (2) memory, (3) visuospatial abilities, (4) selective attention, and (5) general cognitive functioning.

The tertiary outcomes regard psychosocial and physical functioning and include (1) QoL, (2) depressive symptoms, (3) anxiety symptoms, (4) regulation of social and biological rhythms, (5) body awareness, (6) levels of physical activity, and (7) mobility.

Also, the following sociodemographic and clinical variables will be collected for each participant: gender and age, marital status, educational attainment, past and current occupational status, history of and current organic physical illnesses, history of and current mental health diagnoses, and current medication use.

### Evaluation Tools and Timeline

Outcomes will be assessed using validated questionnaires, tests, and performance-based measures at predefined time points (Textbox 1).

**The tools and evaluation procedures.**

**Primary outcomes (feasibility): the tools and evaluation procedures**

- Tolerability, including dropout rates and acceptability, which considers the proportion of recruited participants among those considered eligible. These will be calculated following this timeline: T0 (0 weeks - baseline), T1 (6 weeks), T2 (12 weeks from T1), T3 (24 weeks from T1), T4 (36 weeks from T1), and T5 (48 weeks from T1).
- Side and secondary effects through the Simulator Sickness Questionnaire (SSQ) [30], a self-report questionnaire including 16 items that evaluate the frequency of unwanted effects due to VR technologies, such as nausea, dizziness, headaches, eye strain, etc. This questionnaire will be administered following this timeline: T0 (0 weeks - baseline) and T1 (6 weeks).
- The level of satisfaction with the program as a whole, the effect on psychosocial and physical health, assisting and listening abilities demonstrated by the operators, organizational support for activities, and satisfaction with initial expectations by an ad hoc self-report satisfaction questionnaire. This questionnaire will be administered at T1 (6 weeks after T0).

**Secondary outcomes (cognitive functioning): the tools and the evaluation procedures**

- Trail Making Test [31]: it is a widely administered neuropsychological assessment tool designed to evaluate cognitive flexibility, visual attention, and processing speed. It consists of 2 parts: TMT-A, which requires participants to connect numbered circles sequentially, and TMT-B, which alternates between numbers and letters, assessing more complex executive functions such as task-switching and set-shifting. Reliability studies have reported Cronbach α values ranging from 0.70 to 0.89, depending on the sample characteristics and testing conditions. The questionnaires will be administered following this timeline: T0 (0 weeks), T1 (6 weeks), T2 (12 weeks from T1), T3 (24 weeks from T1), T4 (36 weeks from T1), and T5 (48 weeks from T1).
- Digit Span [32]: it is a neuropsychological test used to evaluate working memory, attention, and immediate verbal memory. It consists of 2 components: Digit Span Forward, which evaluates simple attention and memory span by requiring participants to repeat a sequence of numbers in the same order, and Digit Span Backward, which assesses working memory and cognitive flexibility by requiring participants to repeat the sequence in reverse order. Reliability studies have reported Cronbach α values ranging from 0.70 to 0.90, indicating good internal consistency across different populations and testing contexts. The test will be administered following this timeline: T0 (0 weeks), T1 (6 weeks), T2 (12 weeks from T1), T3 (24 weeks from T1), T4 (36 weeks from T1), and T5 (48 weeks from T1).
- Stroop Test [33]: it is a widely used neuropsychological test that measures cognitive control, selective attention, and processing speed. It evaluates the ability to inhibit automatic responses and resolve cognitive interference by requiring participants to identify the ink color of a word that may denote a different color name (eg, the word “red” printed in blue ink). The test typically includes 3 conditions: “reading colour names,” “naming coloured blocks,” and “naming the ink colour of incongruent colour-word pairs.” The Stroop effect, reflected in the increased time or errors during the incongruent condition, provides insights into executive functioning and attentional control. Cronbach α values reported in reliability studies range from 0.72 to 0.91, depending on the version of the test and the characteristics of the population studied. The test will be administered following this timeline: T0 (0 weeks), T1 (6 weeks), T2 (12 weeks from T1), T3 (24 weeks from T1), T4 (36 weeks from T1), and T5 (48 weeks from T1).
- Rey Figure Test [34]: it is a neuropsychological assessment that measures praxis abilities, visuospatial abilities, working memory, long-term memory, and executive functions. The test involves having a person first copy a complex drawing and then reproduce it from memory after a delay. Studies have reported varying Cronbach α values, but they generally indicate adequate internal consistency for the Rey Figure Test scoring methods. The test will be administered following this timeline: T0 (0 weeks), T1 (6 weeks), T2 (12 weeks from T1), T3 (24 weeks from T1), T4 (36 weeks from T1), and T5 (48 weeks from T1).
- Frontal Assessment Battery (FAB) [35]: it is a brief neuropsychological assessment instrument designed to evaluate executive functions and cognitive processes associated with frontal lobe activity. It consists of 6 subtests assessing different aspects of executive function: conceptualization, mental flexibility, motor programming, sensitivity to interference, inhibitory control, and environmental autonomy. Studies have reported Cronbach α values ranging from 0.78 to 0.90, indicating good internal consistency across various populations. The test will be administered following this timeline: T0 (0 weeks), T1 (6 weeks), T2 (12 weeks from T1), T3 (24 weeks from T1), T4 (36 weeks from T1), and T5 (48 weeks from T1).
- Matrix Test [36]: it is a cognitive assessment tool designed to evaluate nonverbal reasoning, abstract thinking, and problem-solving abilities. It typically involves identifying patterns or completing sequences in visual matrices. The reliability of the Matrix Test, as indicated by Cronbach α, generally ranges from 0.80 to 0.90, reflecting good to excellent internal consistency. The test will be administered following this timeline: T0 (0 weeks), T1 (6 weeks), T2 (12 weeks from T1), T3 (24 weeks from T1), T4 (36 weeks from T1), and T5 (48 weeks from T1).
- Rey Word Test [37]: it is a neuropsychological measure designed to assess verbal memory performance and identify potential malingering through tasks involving the recall and recognition of word lists. The Cronbach α falls within a range of 0.70 to 0.85, depending on the specific population and administration conditions, indicating acceptable to good internal consistency. The test will be administered following this timeline: T0 (0 weeks), T1 (6 weeks), T2 (12 weeks from T1), T3 (24 weeks from T1), T4 (36 weeks from T1), and T5 (48 weeks from T1).
- Addenbrooke’s Cognitive Examination-Revised (ACE-R) [29]: it is a brief tool used for the assessment of 5 key cognitive domains: attention and orientation, memory, verbal fluency, language, and visuospatial abilities. The test has a maximum score of 100, with a recommended range of 66.93-84.93 for the detection of mild cognitive impairment. Notably, ACE-R incorporates the Mini-Mental State Examination (MMSE) as part of its structure. The test will be administered following this timeline: T0 (0 weeks), T1 (6 weeks), T2 (12 weeks from T1), T3 (24 weeks from T1), T4 (36 weeks from T1), and T5 (48 weeks from T1).

**Tertiary outcomes (psychosocial and physical functioning): the tools and evaluation procedures**

- Short Form Health Survey-12 item (SF-12) [38,39]: it assesses the following dimensions of well-being and quality of life: vitality, physical functioning, bodily pain, general health perception, mental, physical, and emotional health, work functioning, and social role. The total score ranges from 12 to 47, with higher scores indicating better quality of life. The test will be administered following this timeline: T0 (0 weeks), T1 (6 weeks), T2 (12 weeks from T1), T3 (24 weeks from T1), T4 (36 weeks from T1), and T5 (48 weeks from T1).
- Patient Health Questionnaire-9 (PHQ-9) [40]: it assesses the presence and severity of 9 depressive symptoms experienced over the past 2 weeks, including depressed mood, anhedonia, sleep and appetite disturbances, low energy, difficulty concentrating, psychomotor disturbances, loss of self-esteem, and suicidal ideation. The total score ranges from 0 to 27, with higher scores indicating greater severity of depressive symptoms. The test will be administered following this timeline: T0 (0 weeks), T1 (6 weeks), T2 (12 weeks from T1), T3 (24 weeks from T1), T4 (36 weeks from T1), and T5 (48 weeks from T1).
- Generalized Anxiety Disorder-7 item (GAD-7) [41]: it assesses the presence and severity of 7 anxiety symptoms experienced over the previous 2 weeks, including nervousness, inability to stop worrying, excessive worry, restlessness, difficulty relaxing, irritability, and fear that something awful might happen. Scores range from 0 to 21, with higher values reflecting more severe anxiety symptoms. The test will be administered following this timeline: T0 (0 weeks), T1 (6 weeks), T2 (12 weeks from T1), T3 (24 weeks from T1), T4 (36 weeks from T1), and T5 (48 weeks from T1).
- Brief Social Rhythms Scale (BSRS) [42]: it assesses the (ir)regularity of rhythms over the past week using 10 items. It evaluates sleep (wake-up and bedtime), eating habits (across different meals), and social interactions (eg, work and/or leisure activities). Scores range from 10 to 60, with higher scores indicating poorer rhythm regularity. The test will be administered following this timeline: T0 (0 weeks), T1 (6 weeks), T2 (12 weeks from T1), T3 (24 weeks from T1), T4 (36 weeks from T1), and T5 (48 weeks from T1).
- Physical Body Experiences Questionnaire Simplified for Active Aging (PBE-QAG) [43]: it assesses body awareness over the past month, focusing on 4 dimensions using 12 items: relationship with one’s own body, body acceptance, awareness of physical abilities, and awareness of physical limitations. Scores range from 12 to 60, with higher scores indicating lower overall body awareness. The test will be administered following this timeline: T0 (0 weeks), T1 (6 weeks), T2 (12 weeks from T1), T3 (24 weeks from T1), T4 (36 weeks from T1), and T5 (48 weeks from T1).
- International Physical Activity Questionnaire (IPAQ) [44]: it assesses the type and amount of physical activity performed over the past 7 days, including both work-related and leisure activities, using 9 items. The total score is based on the conventional unit of measure “MET,” where higher MET corresponds to higher levels of physical activity. The test will be administered following this timeline: T0 (0 weeks), T1 (6 weeks), T2 (12 weeks from T1), T3 (24 weeks from T1), T4 (36 weeks from T1), and T5 (48 weeks from T1).
- Sway area in the presence and absence of visual input [45]: it assesses the quantitative balance maintenance abilities under static conditions. The evaluation will be conducted under 2 conditions: eyes open and eyes closed. Participants will be asked to step onto the platform barefoot and remain as still as possible for 30 seconds. The test will be repeated 3 times for each condition. This assessment will be administered according to the following timeline: T0 (baseline, 0 weeks) and T1 (6 weeks).
- Gait speed analysis (GA) [46]: it assesses the analysis of gait, using a miniaturized wearable inertial sensor. The sensor is positioned on the lumbar region of the trunk at the level of vertebrae L5-S1. The participant is asked to walk at a comfortable, self-selected speed along a straight, unobstructed path of at least 2 m. This assessment will be administered according to the following timeline: T0 (baseline, 0 weeks) and T1 (6 weeks).
- Timed-Up-and-Go (TUG) [47]: it assesses the functional mobility in older adults. The test involves standing up from a chair, walking for 3 m, turning around an obstacle, returning to the starting point, performing a 180° turn, and sitting back down. The participants will perform an instrumented version of the TUG using a wearable inertial sensor placed on the lower back at the level of vertebra L2. This assessment will be administered according to the following timeline: T0 (baseline, 0 weeks) and T1 (6 weeks).

### Data Management and Security

The main goal of the data management process is to guarantee the timely delivery of high-quality data in order to meet the requirements of appropriate statistical analysis. It includes all phases of data management, such as data collection, processing, and application. Clinical data will be compiled into a structured longitudinal database to ensure consistency and facilitate research activities. Each participant will be assigned a unique identification number at the start of the study. The study will be conducted following the Declaration of Helsinki [48] and the requirements of all applicable local and international standards, according to data protection laws.

### Adverse Event Reporting

Each adverse event will be documented with detailed information, including the type of event, onset and resolution dates, severity, expectedness, outcome, and its potential relationship to the study.

### Data Analysis, Statistical Power, and Sample Size

The statistical analysis will use SPSS software (version 21; IBM Corp). Descriptive statistics will be calculated for all variables, with nominal variables reported as frequencies and percentages (n; %) and continuous variables as means and SDs (mean, SD).

Given the feasibility-oriented nature of this trial, all analyses of secondary and tertiary outcomes are exploratory and are not intended to provide definitive evidence of effectiveness. The inclusion of multiple cognitive, psychosocial, and physical outcomes is justified by the need to obtain preliminary effect size estimates and variability parameters that will guide the selection and prioritization of outcomes in a future fully powered RCT. Therefore, the analyses of secondary and tertiary outcomes will be interpreted as hypothesis-generating. No formal power calculations for effectiveness end points have been performed, as the current sample size is intended to assess procedural feasibility and parameter estimation rather than hypothesis testing.

To assess baseline homogeneity between the control group and the intervention group, chi-square tests will be used for nominal variables and 1-way ANOVA for continuous variables. Expected differences related to “group,” “time,” and the “group × time” interaction for all dependent variables will be analyzed using a series of multivariate ANOVA. Specifically, repeated-measures ANOVA and multivariate ANOVA will be applied to continuous variables, while the De Castellan test will be used for nominal variables. Bonferroni correction will be applied where appropriate. The assumption of normality for dependent variables will be examined in relation to sphericity, with the Mauchly test used to assess the equality of variances across related group combinations. Cases with missing data will be treated as incomplete and excluded from the analyses. Secondary analyses may be conducted on specific subgroups (eg, by age) and on selected outcomes (eg, individual items from the administered questionnaires).

Regarding statistical power, it is important to note that no previous RCTs have examined the effects of an immersive VR intervention using virtual sailing scenarios in this population. For this reason, this study is designed as a feasibility and exploratory trial. Although this, the planned sample size is consistent with previous feasibility studies using VR-based paradigms, which have typically enrolled small to moderate cohorts [18,46-48]. On this basis, a total sample of 40 participants (20 per arm) was considered appropriate to assess procedural feasibility, obtain reliable estimates of variability, and capture preliminary effect size signals to inform the design for a fully powered RCT.

### Ethical Considerations

In accordance with the guidelines of the Declaration of Helsinki [48], written informed consent will be obtained from all participants prior to enrollment and data collection. Participants will be fully informed about the aims and procedures of the study and about their right to withdraw at any time without consequences. The study protocol was approved by the Ethical Committee of the “Azienda Ospedaliero-Universitaria di Cagliari,” Italy (Prot. NP/2023/2557, June 16, 2023). All data will be collected and processed in compliance with the European General Data Protection Regulation (GDPR; EU Regulation 2016/679). Data will be anonymized and stored securely, and only the research team will have access to identifiable information. No financial compensation will be provided to participants for taking part in the study.

## Results

This project was initially funded in 2022 for a planned duration of 2 years. A 12-month extension was requested and subsequently granted due to delays in the recruitment and implementation phases. Particularly, recruitment was slowed by post–COVID-19 challenges: given the advanced age and MCI of the target population, regular access to the participating health care facilities and engagement in the study were not always feasible. Furthermore, the intervention was originally designed to be delivered through a sailing navigation simulator based on semi-immersive VR technology, which required a dedicated physical environment. Persistent logistical barriers in securing a suitable setting led the research team to develop and adopt a more advanced immersive VR device, which preserved the original study objectives but required additional time before submission and approval by the Ethics Committee.

Recruitment activities began in May 2024 and are planned to be completed by November 2025. As of manuscript submission, 153 participants were recruited and screened regarding inclusion and exclusion criteria. Among them, 39 participants have been enrolled. Preliminary pre-post analyses are scheduled for January 2026; follow-up analyses will be completed by January 2027.

## Discussion

### Principal Findings

This pilot RCT is expected to provide critical information regarding the feasibility, acceptability, and preliminary signals of effectiveness of an immersive VR-based cognitive remediation program for older adults with MCI. We anticipate that the intervention will be feasible to deliver within routine clinical settings, well tolerated by participants, and associated with improvements in selected cognitive domains, psychosocial functioning, and motor performance. These anticipated findings will guide the refinement of both the intervention and the study procedures for a future fully powered trial.

Over the past decade, the integration of VR as a rehabilitative intervention for older individuals with MCI has shown promising outcomes [20,21,23-26]. Recent meta-analyses conducted on adult individuals with neuropsychiatric disorders, including MCI, demonstrated a positive effect on clinical cognitive outcomes, particularly in the domains of memory, language, and global cognition [25,26]. However, limited evidence is available concerning improvements in clinical and functional outcomes, primarily due to the heterogeneity of variables and assessment tools used, as well as the need for advanced methodological quality in existing studies [20,21,23-27]. Overall, the use of technological innovations and tools such as VR, supported by the existing evidence, can strengthen the rehabilitation experience by increasing engagement and promoting the generalization of acquired skills to real-world contexts, given that the simulated scenarios offer ecologically valid and contextually relevant experiences [18,19,22,49]. While technological tools offer significant support to rehabilitation, they should complement rather than replace the therapeutic relationship, which remains a cornerstone of effective treatment.

The methodological approach adopted in this study reflects a person-centered and recovery-oriented model of rehabilitation, enabling participants to develop their skills and realize a significant improvement in their health and psychological well-being [19,50,51]. Moreover, it adheres to the contemporary framework for the development of complex interventions, wherein ensuring the reproducibility of such interventions constitutes a critical methodological consideration [27]. This study is primarily designed as a feasibility investigation. The findings of this study will also contribute valuable insights that may be translated into clinical practice, particularly concerning the practicability of an innovative therapeutic approach aimed at preventing cognitive decline in older adults with MCI. These findings may carry important practical implications for health care systems, particularly in light of the substantial economic burden associated with cognitive disorders and preliminary clinical effectiveness.

Findings of the present RCT will be able to guide clinical decisions in terms of both the choice of treatment and personalization of the same. The study will ultimately favor the application of safe, noninvasive, and nonpharmacological interventions for the population of older adults with MCI. The systematic monitoring of the intervention phase will support informed decision-making by policymakers regarding the integration of this evidence into standard clinical care pathways.

Strengths of this study include the use of a rigorous feasibility RCT design, standardized VR-based cognitive remediation procedures, multimodal assessment (cognitive, psychosocial, and motor domains), and alignment with current frameworks for the development of complex interventions. The integration of both mental health professionals and biomechanical engineers ensures high-quality implementation and fidelity.

Limitations may arise from recruitment challenges in an older population with cognitive impairment, potential variability in participants’ technological familiarity, and the single-center nature of the trial, which may affect generalizability. As this is a pilot study, it is not powered to detect robust clinical effects; instead, it is designed to generate preliminary signals and inform the next phase of research.

### Next Steps for Scale-Up and Implementation

Should the feasibility indicators and preliminary signals of effectiveness be confirmed, the results of this pilot study will directly inform the design of a fully powered RCT. The subsequent phase of research will focus on optimizing recruitment procedures, refining the intervention dosage and session structure, and further standardizing the delivery of the VR-based cognitive remediation protocol across different clinical settings. Implementation-oriented elements, such as training requirements for health care personnel, logistical needs for VR infrastructure, and preliminary assessments of cost-effectiveness, will also be incorporated to evaluate the scalability and sustainability of the intervention within routine care pathways. Moreover, the findings of this study will support the development of an implementation framework aimed at facilitating the integration of immersive VR-based cognitive remediation into prevention and rehabilitation services. Larger-scale trials will ultimately be required to assess long-term outcomes, generalizability, and health-economic impact.

### Risk and Benefits

The use of immersive VR may be associated with mild and transient side effects, including dizziness, nausea, headaches, visual fatigue, impaired limb coordination, diminished postural stability, a decreased sense of presence, and potentially inappropriate reactions to real-world environments. Nonetheless, significant adverse effects are not expected, as VR is considered a safe and well-tolerated approach, with an extremely low risk of side effects [18,19,22,49-51].

### Dissemination of Results

Findings from this pilot trial will be disseminated through peer-reviewed publications, conference presentations in translational medicine, neuropsychology, and digital health, and stakeholder meetings with local health care professionals and authorities. Additionally, user-friendly summaries will be provided to participants, caregivers, and clinical staff. If the intervention proves feasible and preliminary outcomes are encouraging, dissemination will also target policymakers to support the potential integration of VR-based cognitive interventions into standard care.

### Conclusions

This study will yield clinically translatable insights into the feasibility and preliminary clinical potential of an innovative, VR-based, nonpharmacological cognitive remediation program designed to prevent cognitive decline in older adults with MCI. The findings are expected to inform preventive and rehabilitative practices within health care systems, supporting treatment selection and personalization. By promoting safe, noninvasive strategies, this trial aims to advance the integration of VR interventions into routine care for cognitive decline. Future trials with larger samples will be required to establish efficacy and cost-effectiveness.

## Appendix

Multimedia Appendix 1 Peer review report 1 from Fondazione di Sardegna (Foundation of Sardinia, Italy).

Multimedia Appendix 2 Peer review report 2 from Fondazione di Sardegna (Foundation of Sardinia, Italy).

## Abbreviations

ACE-R: Addenbrooke’s Cognitive Examination-Revised
CONSORT: Consolidated Standards of Reporting Trials
MCI: mild cognitive impairment
MMSE: Mini-Mental State Examination
RCT: randomized controlled trial
QoL: quality of life
VR: virtual reality
VSail: Virtual Sail 3D

## Data Availability

The datasets generated or analyzed during this study are not publicly available due to the European Union’s individual privacy rules, but are available from the corresponding author on reasonable request.
