# Supplementary material for: Enhancing Cognitive Functions in Older Adults With Mild Cognitive Impairment via Virtual Sail 3D: Protocol for a Feasibility Randomized Controlled Trial
Source: JMIR Res Protoc. 2026 Jan 15;15:e85089. doi: 10.2196/85089 (PMC12856399; doi:10.2196/85089)
Supplement: Multimedia Appendix 1 [file resprot_v15i1e85089_app1.pdf]

EVALUATION FORM

Section	Criteria		Maximum score	Assigned score
A. SOUNDNESS OF THE RESEARCH PROJECT IN TERMS OF QUALITY, RESULTS AND COSTS	**Scientific quality, comprehensiveness and clarity of the project**		12	11
	How complete, clear and effective is the project description?	3		
	Does the project appropriately illustrate and critically analyse the state of the art?	3		
	Does the applicant provide solid preliminary data?	3		
	Is the methodological approach adequate and well-integrated?	3		
	**Relevance and potential innovation of the expected results**		5	4
	How original is the project compared to the state of the art? Please, describe which element (e.g. working hypothesis, approaches, methods) of the project you find especially original.	3		
	How significant is the project in terms of impact of the expected results?	2		
	**Suitability of the economic plan**		3	3
	Is the requested grant appropriate and realistic to carry out the activities proposed in the project? Is the cost breakdown appropriately described?			
B. RESEARCH GROUP FEATURES	**Scientific qualification of the Principal Investigator (evaluated even in terms of standard indicators of the scientific performance)**		5	3
	Considering the scientific publications, does the PI provide a competitive track record for the proposed project?	3		
	Has the PI a solid experience to manage the proposed research project and coordinate the team?	2		
	**Quality of the research group members (evaluated even in terms of standard indicators of the scientific performance)**		3	2
	Considering the scientific publications, are the research team members suitable to fulfil their expected role in the project? Does each research team member provide evidence of solid experience within his/her specific research field?			
	**Quality of the research group members in terms of comprehensiveness and complementarity of their expertise**		2	1
	Are the expertise of the research team members and/or the other collaborating units well-integrated? Does each team member contribute to build a solid research group?			
TOTALE			30	24

A. SOUNDNESS OF THE RESEARCH PROJECT IN TERMS OF QUALITY, RESULTS AND COSTS

i. Scientific quality, comprehensiveness and clarity of the project (description of the principal, specific and operational aims; description of the expected results)

How complete, clear and effective is the project description?
(score: 0 ☐ 1 ☐ 2 ☐ 3 ☒)
Does the project appropriately illustrate and critically analyse the state of the art?
(score: 0 ☐ 1 ☐ 2 ☐ 3 ☒)
Does the applicant provide solid preliminary data?
(score: 0 ☐ 1 ☐ 2 ☒ 3 ☐)
Is the methodological approach adequate and well-integrated?
(score: 0 ☐ 1 ☐ 2 ☐ 3 ☒)

The project is clearly described and all elements are included: state of the art, objectives, methods, competences of the participants, expected results. The proponents mention a collaboration with three international institutions, but it is not clear if these institutions will contribute to the present project or what is their role. Actually, the previous experience in the field is not reported, although the scientific publications of the Principal Investigator demonstrate that the rehabilitation effects of sailing have been widely investigated. The use of the virtual simulator was part of a mentioned ERASMUS+ collaboration. This let us to suppose that the team has the competences to carry out the project successfully and to analyse and interpret the results correctly. The time scheduling is reasonable. There are no preliminary data reported, and the proposed project is intended to be a feasibility and exploratory study. To this respect the number of subjects involved and the number of experimental sessions can be considered adequate.

ii. Relevance and potential innovation of the expected results

How original is the project compared to the state of the art? Please, describe which element (e.g. working hypothesis, approaches, methods) of the project you find especially original.
(score: 0 ☐ 1 ☐ 2 ☐ 3 ☒)
How significant is the project in terms of impact of the expected results?
(score: 0 ☐ 1 ☒ 2 ☐)

Within the panorama of virtual reality applications in rehabilitation the use of sailing to promote physical and cognitive improvements is original. Previous studies of the proponents address the problem by making people with mental disability to attend sailing courses and have experience of real sailing on the sea. The proposal of reproducing the sailing experience through the use of a virtual reality simulator aims at making the technique more practical and feasible in usual rehabilitation environments. Since the investment for the equipment and for the training of personnel is not irrelevant, it seems mandatory to test the effects of this application very thoroughly before proposing it for wider application. In particular, it seems that the positive effects are vanishing after 10-12 months from the end of the training. The project plan correctly intends to monitor the trained subjects at 3, 6, 9, 12 months after the intervention. This will be helpful to understand how the different conditions of the subjects can affect the results and then to come out with appropriate indications for this particular rehabilitation treatment.

iii. Suitability of the economic plan

Is the requested grant appropriate and realistic to carry out the activities proposed in the project? Is the cost breakdown appropriately described?
(score: 0 ☐ 1 ☐ 2 ☐ 3 ☒)

The cost of the project is reasonable and it seems appropriate to the purpose.

B. RESEARCH GROUP FEATURES

i. Scientific qualification of the Principal Investigator (evaluated even in terms of standard indicators of the scientific performance)

Considering the scientific publications, does the PI provide a competitive track record within the proposed project?
(score: 0 ☐ 1 ☐ 2 ☒ 3 ☐)
Has the PI a solid experience to manage the proposed research project and coordinate the team?
(score: 0 ☐ 1 ☒ 2 ☐)

The project proposal contains the bibliography of the participants, and it appears that the PI has published 11 articles on referenced journals in the years 2016-2019. Since the bibliography is not reported in a conventional way, it is difficult to access the articles and to ascertain their content. Based on the titles it seems the articles are within the area of interest for the present proposal, so it appears that the PI has the competence to lead the research and to analyze the results in a scientific way. Based on the reported scientific activity, the PI has been contributing to only one research project leaded by the University of Murcia (Spain), founded by the Erasmus+ program, with similar objectives as the present proposal. So, the experience to manage research projects is not demonstrated although it is presumable that, due to the competence in this specific item, and the collaboration of experienced participants, the PI will be able to carry out the project successfully.

ii. Quality of the research group members (evaluated even in terms of standard indicators of the scientific performance)

Considering the scientific publications, are the research team members suitable to fulfil their expected role in the project? Does each research team member provide evidence of solid experience within his/her specific research field?
(score: 0 ☐ 1 ☐ 2 ☒ 3 ☐)

Based on the titles of the reported publications it seems that the different aspects of the project are covered by the team members. This is also confirmed by the complementary experience reported in the individual CV. Unfortunately, the mentioned publications are not easily accessible and so it is difficult to ascertain about their scientific quality. Based on the CV of the participants, it appears that all of them have a consolidated experience in their respective fields. Their specific research fields include: physiology and physiopathology of the physical exercise, measuring ageing and arterial ageing, cardiology and cardiovascular diseases, ageing and mental health, respiration mechanisms, psychology and learning disorders.

iii. Quality of the research group members in terms of comprehensiveness and complementarity of their expertise

Are the expertise of the research team members and/or the other collaborating units well-integrated? Does each team member contribute to build a solid research group?
(score: 0 ☐ 1 ☒ 2 ☐)

There are 6 team members, beside the PI, who are mentioned with their publications and CV. There are three additional participants that are PhD students. The project describes the contribution of three project units: Unit 1- coordination unit; Unit 2- sport unit; Unit 3- geriatric and cardio-pulmonary health unit. It is not clear who of the participants will be included in which Unit. The distribution of the tasks to the research units is rather generic and does not mention the location and the facilities required, for example: where the subjects will be contacted and recruited, where the equipment will be installed, where the blood samples will be collected and then analysed, who will analyse the different types of data. It can be presumed that the whole team will cooperate on several aspects, but the lack of a prior assignment of tasks raises a question about the smooth development of the project.

OVERALL COMMENTS AND SUGGESTIONS

The project is interesting and original. The use of a sailing simulator to improve the condition of elderly people with mild cognitive impairments has a potential in rehabilitation and is worth investigating. The study protocol is relatively comprehensive and well described. The weak points are not referred to the project content but to the form of presentation: the bibliography of the team

members is referenced in no-conventional way (the iris/UNICA code instead of authors, title, year, journal) and thus it is impossible to access the articles and ascertain the quality of the publications; the role of the different members is not described, nor is their participation to the three project units; the logistic and organizational aspects are not mentioned (where the simulator will be installed, where the blood samples will be collected and analysed, how the different phases of experimental training, testing, data collection, data processing will be organised…). These shortcomings make it difficult to assess the real feasibility of the project, which appears sound from the point of view of the contents.
