# Supplementary material for: Enhancing Cognitive Functions in Older Adults With Mild Cognitive Impairment via Virtual Sail 3D: Protocol for a Feasibility Randomized Controlled Trial
Source: JMIR Res Protoc. 2026 Jan 15;15:e85089. doi: 10.2196/85089 (PMC12856399; doi:10.2196/85089)
Supplement: Multimedia Appendix 2 [file resprot_v15i1e85089_app2.pdf]

EVALUATION FORM

Section	Criteria		Maximum score	Assigned score
A. SOUNDNESS OF THE RESEARCH PROJECT IN TERMS OF QUALITY, RESULTS AND COSTS	**Scientific quality, comprehensiveness and clarity of the project**		12	9
	How complete, clear and effective is the project description?	3		
	Does the project appropriately illustrate and critically analyse the state of the art?	3		
	Does the applicant provide solid preliminary data?	3		
	Is the methodological approach adequate and well-integrated?	3		
	**Relevance and potential innovation of the expected results**		5	3
	How original is the project compared to the state of the art? Please, describe which element (e.g. working hypothesis, approaches, methods) of the project you find especially original.	3		
	How significant is the project in terms of impact of the expected results?	2		
	**Suitability of the economic plan**		3	3
	Is the requested grant appropriate and realistic to carry out the activities proposed in the project? Is the cost breakdown appropriately described?			
B. RESEARCH GROUP FEATURES	**Scientific qualification of the Principal Investigator (evaluated even in terms of standard indicators of the scientific performance)**		5	5
	Considering the scientific publications, does the PI provide a competitive track record for the proposed project?	3		
	Has the PI a solid experience to manage the proposed research project and coordinate the team?	2		
	**Quality of the research group members (evaluated even in terms of standard indicators of the scientific performance)**		3	3
	Considering the scientific publications, are the research team members suitable to fulfil their expected role in the project? Does each research team member provide evidence of solid experience within his/her specific research field?			
	**Quality of the research group members in terms of comprehensiveness and complementarity of their expertise**		2	1
	Are the expertise of the research team members and/or the other collaborating units well-integrated? Does each team member contribute to build a solid research group?			
TOTALE			30	24

A. SOUNDNESS OF THE RESEARCH PROJECT IN TERMS OF QUALITY, RESULTS AND COSTS

i. Scientific quality, comprehensiveness and clarity of the project (description of the principal, specific and operational aims; description of the expected results)

How complete, clear and effective is the project description?
(score: 0 ☐ 1 ☐ 2 ☒ 3 ☐)
Does the project appropriately illustrate and critically analyse the state of the art?
(score: 0 ☐ 1 ☐ 2 ☒ 3 ☐)
Does the applicant provide solid preliminary data?
(score: 0 ☐ 1 ☐ 2 ☒ 3 ☐)
Is the methodological approach adequate and well-integrated?
(score: 0 ☐ 1 ☐ 2 ☐ 3 ☒)

The project proposal is generally well-written and clear, providing a sufficiently detailed picture of the project's objectives and the methodologies to pursue them.
The main references from the state of the art are properly listed and the project's objectives tend to be fairly contextualized in the literature. However, some of the claims are not fully supported. For example, in a passage authors say that literature trials are characterized by "unclear or contradictory indications on timing and optimal modality of administration". This is a strong statement that should be supported ideally by a review of recent works or by, at least, some recent examples that represent such type of limitation. On the same line, it is said that the proposed study is based on a semi-immersive VR setting which was developed in a previous project ("Sail4PDP"). No sufficient details about prior work is provided here, this ultimately makes it difficult to assess what original contributions the present proposal is putting forward and what lessons learned are instead exploited and leveraged.
No preliminary data are provided, but I don't think that this undermines the overall value of the proposal.
The methodological approach is adequate even if some parts could be improved, especially in the way they are described. For example, I found the term *feasibility* not fully pertinent for what the proposal aims at doing. What is seems to emerge from the proposal is that the goal is to assess if the administered training session will be effective in improving a list of (primary and secondary) indicators related to subject's health. I am not sure if this is completely in line with a feasibility study which, instead, should focus on achieving a convenient (i.e., with affordable costs per patient or installation) and scalable way to organize and administer the sessions as well as gathering, analysing and using the results. Similarly, the proposal is not outlining the challenges of recruiting the 60 subjects (a number whose management is not straightforward) apart from a pre-set dropout rate.

ii. Relevance and potential innovation of the expected results

How original is the project compared to the state of the art? Please, describe which element (e.g. working hypothesis, approaches, methods) of the project you find especially original.
(score: 0 ☐ 1 ☐ 2 ☒ 3 ☐)
How significant is the project in terms of impact of the expected results?
(score: 0 ☐ 1 ☒ 2 ☐)

The project's places itself in a well-established line of research, which is focused on using exergaming technology for active and healthy aging and that inspired a large body of literature and funded projects in different scientific communities. With respect to this body of research, the main novelty of the proposal seems to reside in the employment of a very specific and advanced VR setup, which probably cannot be ascribed to the typical standard platforms (present on the market or deployed in specific settings). Under this perspective, the potential impact of the project can be considered narrow in its domain of influence, despite the more general research question is without any doubt a significant one. The proposal draft does not provide a solid and convincing argumentation about novelty and impact of the specific investigation that is outlined. The reader is convinced that the general domain of applying exergaming technology to active aging is prominent, full of challenges and characterizing by promising results already present in the state of the art. But a discussion around these issues in the very specific scope of sailing simulation for active aging is not fully developed.

iii. Suitability of the economic plan

Is the requested grant appropriate and realistic to carry out the activities proposed in the project? Is the cost breakdown appropriately described?
(score: 0 ☐ 1 ☐ 2 ☐ 3 ☒)

The requested grant seems more than appropriate for the type of activities that are envisioned in the project proposal. There are parts, however, that could be improved. For example, risk analysis, exploitation, and communication are not covered by the budget planning. Also, the costs declared for the training of the simulator experts are difficult to judge since few details are provided about the specific kind of activities they should carry out (are there any sessions that could be conducted from remote? does training comprise the installation of the setup? is maintenance at any time/level taken into account?)

B. RESEARCH GROUP FEATURES

i. Scientific qualification of the Principal Investigator (evaluated even in terms of standard indicators of the scientific performance)

Considering the scientific publications, does the PI provide a competitive track record within the proposed project?
(score: 0 ☐ 1 ☐ 2 ☐ 3 ☒)
Has the PI a solid experience to manage the proposed research project and coordinate the team?
(score: 0 ☐ 1 ☐ 2 ☒)

The PI shows a clear and solid research record in the related field. The scientific production is, to the best of this reviewer's knowledge, competitive and of quality. The scientific production is steady and in line with the primary scientific challenges of the proposed project. Particularly interesting is the fact that the PI has already some experience with this type of technology and with the very same setup that will be used in the project.
The PI's cv shows a number of past collaborations also involving funded projects in similar contexts. Project coordination experience does not starkly emerge from the proposal, but other records clearly show that the PI would be more than suitable to coordinate a project of this scale.

ii. Quality of the research group members (evaluated even in terms of standard indicators of the scientific performance)

Considering the scientific publications, are the research team members suitable to fulfil their expected role in the project? Does each research team member provide evidence of solid experience within his/her specific research field?
(score: 0 ☐ 1 ☐ 2 ☐ 3 ☒)

The research group's record and publications clearly show how each member has a well-established research experience in his/her respective field. Areas of expertise well match the primary requirement of the project, with particular focus on the medical scope. The set of profiles of the teams seems more than suitable for an effective implementation of the project's activities. All members have a good publication record and/or past experience in projects collaboration/supervision which add value to the consortium. The presented list of publication outlines a good rate of scientific productivity which, to be best of this reviewer's knowledge, is of good quality.

iii. Quality of the research group members in terms of comprehensiveness and complementarity of their expertise

Are the expertise of the research team members and/or the other collaborating units well-integrated? Does each team member contribute to build a solid research group?
(score: 0 ☐ 1 ☒ 2 ☐)

Considered in their ensemble, the research team is characterized by a set of skills that exhibits good complementarity in relation to those primary objectives of the project related to the medical area. The types of roles, competences, and past experiences support the possibility for a smooth and fruitful collaboration. The team is also characterized by a good gender balance.
The project proposal, however, does not provide a clear description of what tasks and responsibilities each group member shall take on in the carrying out of the project. So, while from a general point of view is rather clear that the team members are more than suitable to effectively supervise and administer the project's activities, from a more specific perspective is not quite clear who is going to do what. Another shortfall is that technical competences (in the development or usage of VR technology) seem to be mainly externalized.

OVERALL COMMENTS AND SUGGESTIONS

The project pursues a scientific goal framed inside a domain of great general interest: the application of VR exergaming technology (specifically, a semi-immersive VR experience based on a sailing simulator) to active aging. The methodology proposed to carry out the project is, apart from some minor shortfalls (see comments above), sound and appropriate. The research team seems to be more than suitable to tackle the research challenges and goals described in the proposal.
From the other side, the project's application domain of investigation is quite narrow and limited to a very specific platform whose choice is not fully justified and detailed in the proposal. A big picture of an envisioned scenario is missing. For example, is not clear how such sessions would be administered in a fully deployed future setting (At home? At specific facilities? Within what program? With what kind of clinical/technical assistance? With what costs and required level of commitment from the patient?). This must be addressed having in mind that the literature also proposes solutions for active aging which are transparent, low cost, and deployed in the domestic environment. The type of PA (Which parts of the body are involved? Which level of difficulty can be set? Which physical impairments do not allow the use of the setup?) that the simulator allows to carry out is not described in detail and, without having a full technical description of the simulator, are difficult to infer. The platform is referred as 2D, but this is terminology is seldom used in the VR community (semi-immersive, for example, would be better). Also, it is claimed about extensions to the 3D domain without providing any further discussion in the document. All these issues deeply impact feasibility, but the proposal seems more concerned with health-related indicators that measure the potential effectiveness of such type of training activity. Against this background, the proposal does a fair job in describing the general scope and the implementation details of the research activities, but lacks a strong motivation to support impact and significance beyond the specific setting tackled in the project.
